# Current Methods and Technologies for Storage Tank Condition Assessment: A Comprehensive Review

**DOI:** 10.3390/ma18051074

**Published:** 2025-02-27

**Authors:** Alexandru-Adrian Stoicescu, Razvan George Ripeanu, Maria Tănase, Liviu Toader

**Affiliations:** 1 Mechanical Engineering Department, Petroleum-Gas University of Ploiesti, 100680 Ploiesti, Romania; alexandru-adrian@upg-ploiesti.ro; 2 Technical Lead & Development Srl, 107063 Corlatesti, Romania; liviu.toader@tld-romania.com

**Keywords:** storage tank, condition assessment, equipment lifetime, oil storage, lifetime extension, non-destructive testing

## Abstract

This study investigates the current industry practices for storage tank assessment and the possibilities for improving inspection methods using the latest technologies on the market. This article presents the main methods and technologies for non-destructive testing (NDT), along with new methods that make them more efficient and economical. To further analyze the state of a tank and determine its lifetime expectancy, analysis methods are presented based on NDT results. The key aspects that can be improved and made more efficient are NDT procedures using robots/drones and autonomous devices; automated inspection procedures, like remote video inspection combined with local thickness measurement or 3D scanning of the tank elements for deformations; advanced analysis methods using the input from the NDT and inspection data collected using analytical calculations according to applicable standards; Finite Element Analysis (FEA); and digitalized models of equipment (Digital Twin) accompanied by artificial intelligence for data processing. The best way to make the process more efficient is to develop and use dedicated standardized software for tank condition assessment.

## 1. Introduction

Aboveground storage tanks (ASTs) are used in almost all industrial applications where large liquid substance storage is a part of the process. The most important application is the storage of hydrocarbon and petrochemical products, which are made in welded, vertical, cylindrical aboveground storage tanks.

ASTs have the three following main components: the bottom, the shell, and the roof. Additionally, ASTs have nozzles for process, instrumentation, and firefighting connections, steel structures for personnel access, and various internal aspects, depending on the storage application (internal floating roof, still pipes, heating coils, inlet diffusers, floating suction arms, etc.). In regard to equipment definition (battery limit), the tank itself includes the assembled bottom, shell, roof, and nozzles, which have strict rules of calculation, design, and fabrication, with the rest being auxiliaries that are also part of the tank but do not play a structural role. This is the most used model in the industry. The reason is that some auxiliaries, like access structures, heating coils, etc., have totally different design and fabrication standards, and the only thing that should be considered in the main tank assembly regarding auxiliaries is their weight.

It is of the highest importance that tanks are designed and built considering all operational and environmental aspects for a defined lifetime usually of 30 years. During an AST’s lifetime, periodic inspection activities are conducted, and if needed, local revamping activities are completed.

After a long period of operation of more than 30 years, even if a tank does not have any leaks, a thorough condition assessment needs to be completed in order to extend its lifetime. The main issue is corrosion, which affects certain sections of the tank, mainly the bottom [1], but other local defects may endanger tank integrity, like local deformations, weld defects, corrosion under insulation, etc.

This approach is advantageous from many points of view, such as the following:

- Economical: If the tank is in good technical shape, then, maybe with minor revamp works, it could be put back into safe operation and its lifetime could be extended. If the condition assessment conclusion is that the tank can be repaired and the repair works cover a small percentage of the tank, then the cost of a tank revamp would be a fraction of the cost of building a new one.
- Availability: Most of the time, revamping work takes less time than building a new tank, so the equipment can be available in a shorter time.
- Safety: A thorough inspection and condition assessment of a tank that has been in operation for a long time might highlight hidden defects that could cause a catastrophic failure of the tank with environmental, economic, and even human life loss impacts. An example is the accident that took place in Buncefield oil storage depot, NE London, England, on 11 December 2005. It took 5 days to extinguish the fire, resulting in 43 injuries, air and groundwater pollution, depot destruction, and around GDP 700 million of damages [2].

Tank condition assessment is performed following certain standards, like API653 [3] and EEMUA 159 [4]. Since the first editions of the inspection and repair standards (in the late 1980s, following several AST failures in the USA), more improved inspection and testing technologies have been developed, resulting in more reliable condition assessment. If 30 or 40 years ago, only visual inspection and weld NDT were performed to decide if the tank lifetime would be extended, now there is a complex process, including a list of tests and assessments leading to the decision of whether to extend the tank lifetime and with what revamp work. The current technologies allow for full wall thickness measurements of the tank roof/shell/bottom by ultrasonic scanning or similar methods, automatic weld ultrasonic inspection, and 3D LIDAR scanning of the shell and bottom for local deformations. In certain cases, drones are used to take wall thickness measurements, make visual inspections or scans in inaccessible areas, and even to eliminate the economic and schedule impacts of using scaffolding and human-operated equipment. In addition to these standard methods, recent advancements in digital technology have allowed for the development of predictive maintenance models based on real-time monitoring and data analytics. These technologies employ sensors and IoT-enabled devices to continuously monitor the condition of tanks, providing early warning signals for potential defects before they become critical issues. Machine learning algorithms can analyze large sets of historical data and provide predictive insights, reducing the uncertainty associated with manual inspections and helping operators make decisions regarding maintenance and repair strategies.

On the other side, condition assessment studies can be conducted using advanced software tools developed specifically for the analysis of the tank elements. An experienced engineer can interpret the results of NDT and prepare an input for a software tool that can generate a complete report of the tank status. Some of these software programs have Finite Element Analysis (FEA) modules that can analyze the tank assembly. By simulating different loading conditions and failure scenarios, these tools allow for a comprehensive understanding of the structural integrity of a tank and aid in making informed decisions regarding maintenance and repair actions.

If the necessity of the condition assessment of ASTs that have exceeded their initial lifetime or gone through an abnormal operation scenario (like overfill, a local deformation, a leakage, or local corrosion) or even a natural phenomenon, like an earthquake, hurricane, etc., has been highlighted, it is also very important to acknowledge and include the latest inspection and assessment technologies that are available and which continuously evolve.

This article highlights the optimal storage tank condition assessment activities in the European Union legislative context, where dedicated API standards are not always recognized, as they are not mandatorily applicable, creating a bridge with the applicable EU design standards exemplified for a piece of existing equipment while highlighting new methods and trends for these activities. The proposed method can be easily implemented, with optimal cost/time efficiency and, most importantly, following applicable standards.

## 2. Storage Tanks Failure Mods

### 2.1. Materials

Depending on the application field, storage tank materials can vary from the most common carbon steel to stainless steel or nickel-based steel. For different applications, plastic, fiber glass/composite, and concrete storage tanks can be used, but these are outside the scope of the present article.

Material selection is performed by a mechanical engineer based on the characteristics of the stored fluid, especially its corrosivity and storage temperature. For the most common metallic storage tanks, there are two categories of materials, as follows:

- Carbon steel (CS): For low-corrosive products (crude oil, mineral oils, fuels, process water, etc.). The most common examples of CS used are API (ASTM A36, ASTM, A283 Gr. C and D, ASTM A285 Gr. C, and ASTM A516 Gr. 55, 60, 65, and 70) and European (S235, S275, and S355 JR or J2). In some cases, carbon steel can be protected with special liners, like epoxy, polyurethan, and glass, to prevent the stored fluid from coming in contact with the base material [5].
- Stainless steel: For high-corrosive products, like acids, ammonia, etc., or when the product should not be contaminated by any corrosion (potable water, high-oxygen products, special jet fuels, etc.). The most common materials in this category are AISI 304L, AISI 316L, and AISI 316Ti.

All design and fabrication standards have a section for materials selection, depending on the MDMT [6,7], which is calculated in advance.

The present article focuses on carbon steel tanks, as they are the most commonly used in the oil and gas sector [8]. The structural steels S235/S275/S355, or their equivalents, are the most used materials for crude oil storage tanks at ambient temperature.

### 2.2. Typical Degradation Mechanisms and the Influencing Factors

Within a tank’s lifetime, there are several phenomena that can negatively affect its structural integrity.

The main degradation mechanisms are as follows:

- Corrosion, caused by stored fluid and environmental action, which can lead to c content loss and sometimes tank collapse.
- Deformation, which can be caused by auxiliary equipment failure, operating errors, fabrication defects, etc., and can lead to tank instability, content loss, and even collapse.
- Different design/fabrication defects that were not identified during tank erection and have intensified during operation (weld defects, improper design, etc.).
- Extraordinary environmental incidents, which are not usually taken into account, but are unlikely to occur, such as earthquakes, winds, snow, explosions in tank’s vicinity, etc.

Degradation is most commonly caused by corrosion [9]. There are several types of corrosion that can occur within the metallic components of a storage tank.

A brief classification can be made as follows: internal and external corrosion, including carbon dioxide and hydrogen sulfide, pitting, microbial corrosion, corrosion under insulation [8], and differential aeration of the tank shell’s inner side [10]. The main corrosion type in crude oil storage tanks seen during internal inspection is pitting and/or fatigue cracking [11], frequent on the bottom [12], bottom-to-shell weld, and upper side within the vapor space. Bottom corrosion forms due to water accumulation, while upper corrosion appears due to water vapors that condensate on the tank’s upper courses [13].

Another damaging mechanism is deformation, caused by various reasons, such as breathing/safety equipment failure, fabrication defects (in older tanks, when the construction standards were not so rigorous), local deformations caused by wind pressures [14], buckling caused by previous seismic events [15], local deformations caused by excessive hydrostatic pressures [16], and deformations due to foundation settlement [17].

**Figure 1:**
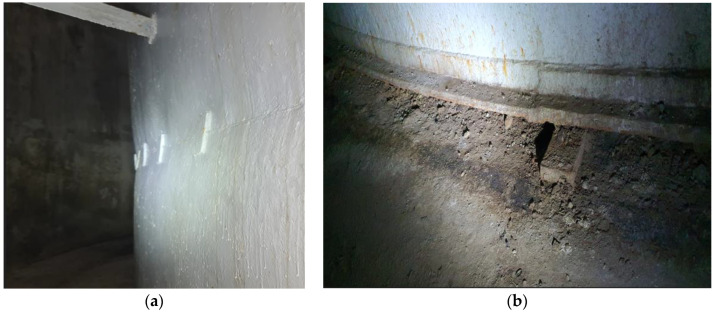
Shell plastic deformation (**a**) and tank bottom lifting off foundation due to elastic bed settling (**b**).

**Figure 2:**
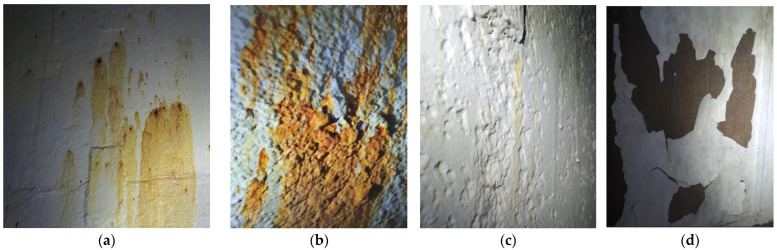
Corrosion protection system damage: external paint damage (**a**,**b**); pitting damage (**c**); and internal lining damage (**d**).

A brief classification of the human factors that can influence tank degradation is as follows:

- Improper operation (loading/unloading at a flow rate higher than the design capacity of the breathing/safety valves) can lead to plastic deformation of the shell [18] (Figure 1a) and bottom, lifting off the foundation [19] (Figure 1b) and even resulting in tank collapse.
- Changing the intended use without equipment modifications, mainly regarding the stored product, can lead to the following:
  - Excessive corrosion (Figure 2), beyond the designed limits, as outlined in Section 3.2, Section 3.3, Section 3.4. This can occur due to the storage of a product more corrosive than the original one (potentially with a high water content or stronger corrosive agents such as H_2_S, CO_2_, H_2_SO_4_, etc.), a reduced frequency of loading/unloading operations, and the formation of condensation, among others.
  - Deformations or even shell failure, if a product with a higher density is stored.

- Improper maintenance, especially of the external/internal anti-corrosion coatings, any sacrificial anode protection systems, fire protection systems [19], and the tank’s protective components (breathing/safety valves).
- Detail design or fabrication deficiencies, usually due to improper mechanical strength calculation, and deficiencies in material selection and anchorage.

## 3. Condition Assessment Techniques

### 3.1. Tanks’ Non-Destructive Inspection

**Figure 3:**
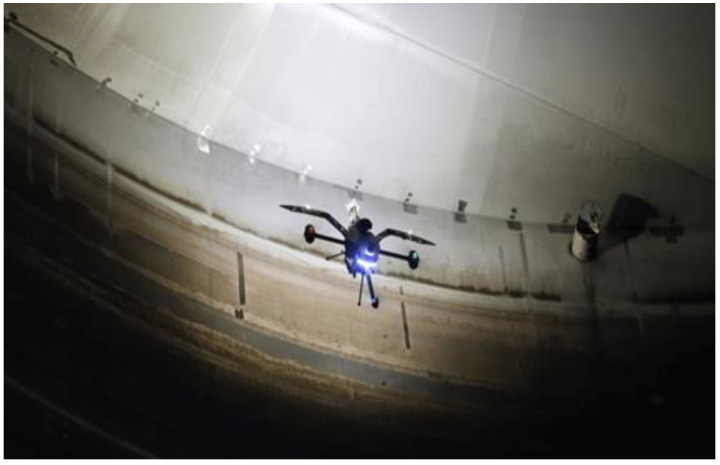
Drone for ultrasonic wall thickness measurement.

The first step of a tank inspection is represented by visual inspections of the main components (bottom, shell, roof structure and plates, nozzles, and other accessories). Visual inspection should be performed both on the outside and the inside of the tank. Usually, to access the upper tank sections, scaffolding is used. It is known that full scaffolding for a large tank represents a serious cost, so modern techniques can be used, like drones (Figure 3) equipped with high-resolution video cameras. Also, these drones can have ultrasonic wall thickness measurement systems that can take thickness measurements of the tank’s upper courses [20,21].

Following these visual inspections, ultrasonic wall thickness measurements for all tank components are conducted (at least five measurements per plate for the tank shell [3]).

As mentioned above, wall thickness measurements can be performed by qualified operators, or by unmanned equipment, like drones. Another procedure can be floor or shell scanning using the SLOFEC technique [22], with the output being a continuous plate measurement that can highlight any thinning, material loss, or fault.

As these tanks are designed with a perfect cylindrical shell and flat bottom, a deformation analysis should be performed to check that the tank’s main components, especially the shell and bottom, are not deformed beyond the limits allowed by standards [3]. The best and most economical procedure is the 3D scanning of a tank, recommended to be performed from inside. Tank deformation scanning with the LIDAR technique [23] can be afterward transposed into a developed tank shell on which any deviation from the theoretical cylindrical shape (that exceeds the allowable value) is highlighted.

A significant percentage of tank leakages or even failures is represented by welding faults, so a comprehensive welding inspection needs to be performed [3]. Welding non-destructive testing procedures are well known and can be conducted by various methods, like ultrasonic testing (UT), magnetic particle testing (MPT), acoustic emission testing (AE), a dye penetrant test (PT), soap bubble examination [6], radiographic testing (RT), etc. [8].

Each design standard has a dedicated section for inspection and testing activities. Both European and API/ASTM standard requirements are similar in this regard. These activities can be of several types, as follows:

- Inspections of the bulk and prefabricated materials;
- Inspection of plates/fittings edges and other items before welding;
- The NDT of welds;
- Dimensional imperfection measurements;
- Erected equipment final tests, like Hydrotest/Pneumatic tests and settlement tests;

Depending on the corrosion protection requirements, additional metal surface preparation and corrosion protection inspection can be performed. For these activities, dedicated standards and the manufacturer’s requirements for corrosion protection materials should be followed.

AST weld seams, and especially their heat-affected zones (HAZs), are susceptible to welding defects and must be checked using the applicable NDT methods.

Depending on material, plates thicknesses, and weld types, the following methods are used:

- Radiographic testing (RT): used for plates with a thickness less than 13 mm and butt-weld nozzles, and as an option for plates with a thickness between 13 mm and 30 mm;
- Ultrasonic testing (UT): used for plates and butt-weld nozzles, as an option for plates with a thickness between 13 mm and 30 mm, and mandatorily for thicker plates;
- Magnetic particle testing (MPT): used for both fillet-welds and butt-welds, but mainly on the bottom and roof, where RT and UT cannot be applied.
- Dye penetrant test (PT): used for welds that are not critical for AST structural integrity (supports attachment plates, clips, etc.);
- Soap bubble examination or vacuum box: used mainly for bottom and roof seal welds.

The extent of testing is specified in each design standard for each weld type.

The personnel that perform the NDT should be certified for the respective method.

Any weld defect should be repaired or the respective element/plate should be replaced.

### 3.2. Analytical Method According to Available Standards and Recommended Practices—Case Study Tank M6

The analytical method for existing storage tank condition assessment represents the first step in investigating the actual equipment’s technical state. Calculation algorithms are governed by the applicable standards, in this case, the standard [3].

The analytical model follows the standard assessment calculation steps. For the ease of mathematical calculations, a custom Microsoft Excel 360 spreadsheet is used. There are also additional dedicated software tools that can be used, like Mathcad or Matlab, but since the calculations are not very complicated, Excel is the best option.

For the calculation purposes, a storage tank that was assessed in the middle of 2024 and was part of a storage facility within a refinery in Romania was considered.

The Tank M6 data are as follows:

- Service: LCO storage;
- Capacity: 10,000 m^3^;
- Diameter: 32.314 m;
- Shell height: 12.97 m (9 courses);
- Operating temperature: 40 °C;
- Operating pressure: 10 mbarg;
- Roof type: conical, auto-portant, truss steel structure;
- Material: S235J2 for bottom/shell/roof plates and S275J2 for structurals.

The input data for these calculations are the measurements made on-site, like the height of the tank and height of each course, the diameter, wall thickness measurements for each component (i.e., each plate at least five points, nozzle necks, roof structural elements, stiffening rings, etc.), anchorage details, weld types, etc. Also, operating/process data (temperature, pressure, specific weight of the stored fluid, etc.) and environmental data (snow, wind, and seismic loads) are considered in the condition assessment calculations.

A calculation was made for Tank M6 (Table 1) according to the requirements of standard [3]. 

The corrosion rate (measured in mm/year), which represents the plates’ material (in direct contact with the stored fluid) thickness loss in one year, is directly dependent on the stored fluid. Finally, the corrosion allowance (CA) is the additional plates’ wall thickness, which needs to be considered with the minimum resistance plates’ thicknesses at the end of the equipment’s lifetime. The CA is obtained by multiplying the corrosion rate by the equipment lifetime. The bottom and shell (which are in contact with the stored fluid) corrosion rate for a carbon steel oil storage tank is usually 0.1 mm/year.

In addition to these standards, depending on the local legislation, other standards can be used, but for verification purposes. In this case, [6,24] are mentioned, which could be used for the verification of a tank’s stability, but only if the tank deformations are within the standard [3] allowable limits (so the cylindrical shape can be considered) and there are no significant local damages to the tank components (like containment breaches, loss of material, punctures due to corrosion, missing or damaged structural elements, etc.). For this verification, an additional corrosion allowance should be considered based on the tank’s corrosion history during its lifetime. The requirements of standard [6] were followed to calculate the values presented in Table 2, while the standard [24] was used for Table 3.

**Figure 4:**
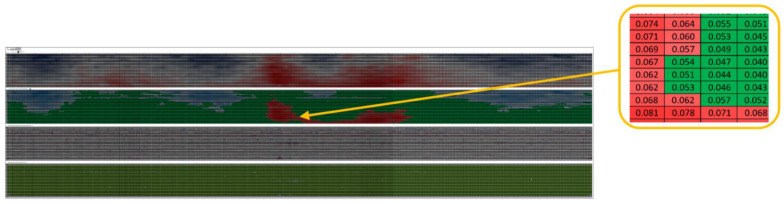
Tank M6 shell deformation map.

For plate sections that are deformed beyond the limits allowed by [3], usually, the only repairment method is replacing the respective plate/area with a new one, whose thickness is determined by calculation. Shell deformations are provided by a specialized expert who measures the deformations by the LIDAR method, with the output being the deformation map presented in Figure 4, for a tank that was assessed in 2024 according to the requirements of standard [3] (Tank M6).

There are several pieces of information within the deformation maps, but the most important data are represented by the numeric values of deviations from a cylindrical shape, in this case, 57 mm, as per [3]. To obtain a clearer picture of the tank shell situation, values that exceed the maximum allowable ones have been highlighted in red for outer deviations and blue for inward deviations. The more stringent the color, the higher the deviation. Values were considered for a plate section of 200 mm × 200 mm.

**Figure 5:**
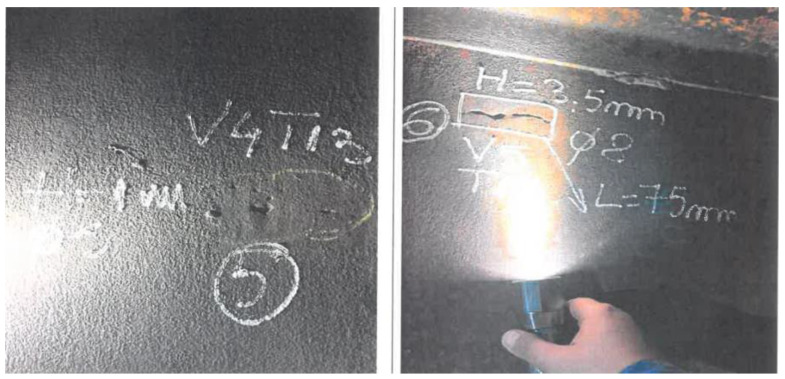
Tank M6 local defect found during visual inspection.

Along with deformations, some defects found during the visual inspection were considered, as presented in Figure 5.

### 3.3. Computational Method for Tank Condition Assessment

There is a vast software availability for both the engineering and condition assessment of various types of equipment, piping, structures, etc.

For the particular case of ASTs, there are few software solutions, like Hexagon Tank^®^, AMETank^®^, and Bentley Autopipe Vessel^®^. The choice depends on the company and the software selection of the engineer who performs the analysis. For the Tank M6 exemplified in this article, Hexagon Tank^®^ was used. This software performs a full analysis according to the requirements of [3] (both static and dynamic analysis).

Following the calculation, the most critical point was hoop stress, as per the requirements of [3], which highlighted that several courses were unfit for safe operation, as presented in Table 4.

Apart from the shell, roof, and bottom, there is an additional critical element that can cause major incidents. This is the tank roof, which can, in some instances, be the weak point of a tank. Tank roof failure can have a series of causes, as follows:

- Steel structure corrosion/damage;
- Overpressure/vacuum due to improper operation or the failure of breathing/emergency equipment (like the incident of an 8 m diameter tank in which the roof was propelled 25 m away from the tank location) [25];
- Cover plates with extensive corrosion damage.

While for the roof cover, the measured plate thicknesses are compared to the minimum allowed by the standard [3], in the case of roof supporting structures, there is a more delicate matter that needs to be assessed separately and, most importantly, according to the applicable regulation of the country where the tank is located. In Romania, tank roof supporting structures are treated like structural elements and fall under the applicable European regulation [26].

In this regard, a structural analysis was performed on the corroded structural assembly of the tank roof supporting structure, using the beams thicknesses measured on-site. A corrosion allowance of 1 mm was applied to all members.

A dedicated structural design software solution should be used for this task. There are multiple software solutions available, and for the case study of Tank M6, Bentley RAM Elements^®^ was used.

The roof of Tank M6 was replaced 10 years previously (but unfortunately without a proper calculation), the revamp detail drawings were available, and the structural modeling went smoothly.

The loads and their combinations were considered as per [26] (wind, snow, seismic, dead load, etc.), along with the operation loads, especially the vacuum/overpressure.

**Figure 6:**
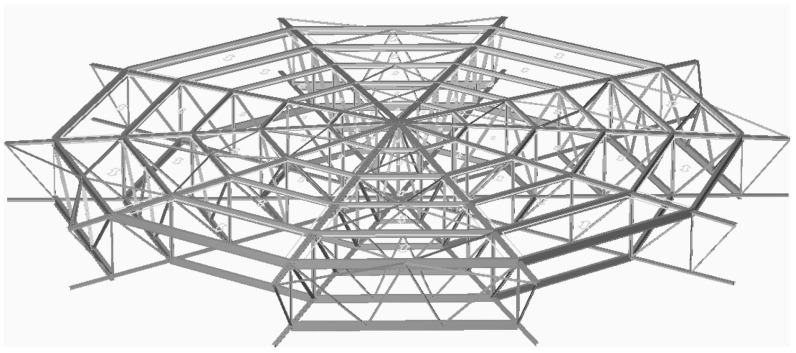
Tank M6 roof structural assembly.

The final model of Tank M6’s structural assembly is shown in Figure 6.

**Figure 7:**
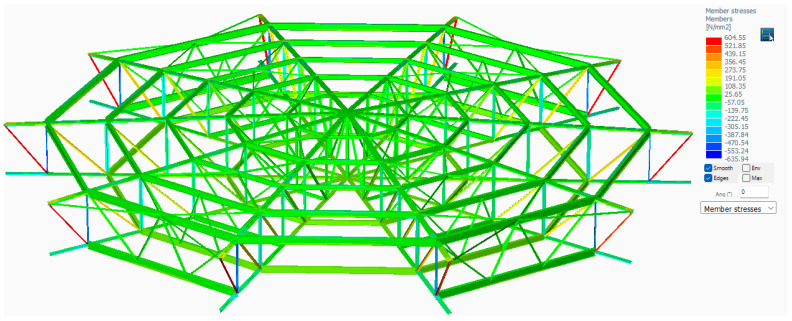
Tank M6 roof structural assembly stress diagram for D6 loads combination.

The most stringent load combination is D6 = 1.35 × Dead Load (including Operating) + 1.05 × Live Load + 1.5 × Snow Load. It can be seen in Figure 7 that the allowable stress of 181.5 MPa (corresponding to S275J2 material after applying the coefficient of 0.66) is overpassed by a very high value of 604.55 MPa.

In this case, it can be concluded that the roof tank supporting structure is unable to be further used in safe operating conditions and a replacement is needed.

As an alternative to the classic carbon steel roof, there are also other roof types that have multiple technical and economic advantages, like aluminum dome roofs.

The advantages of aluminum dome roofs are as follows:

- Lighter weights (sometimes five times lighter than the same roof in carbon steel)
- Ease of construction (structural aluminum beams and sheets can be assembled by bolts and screws on-site);
- No corrosion protection needed (aluminum construction);
- Higher sun reflection capacity (very efficient for volatile product storage);
- High venting capacity.

There are also some disadvantages, as follows:

- Limited usage for higher internal pressure tanks;
- Difficult shell-to-roof sealing;
- Necessity to use an internal floating roof (IFR) for highly volatile product storage (benzene, toluene, xylene, and even gasoline).

Under these considerations, for Tank M6, together with the owner, it was decided to install an aluminum dome roof without an IFR (because the tank was supposed to be used for LCO storage).

**Figure 8:**
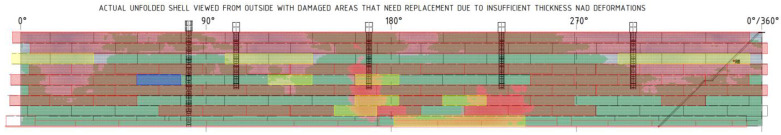
Tank M6 shell map representing: hatched in red the shell plates to be replaced due to insufficient thickness, in yellow the ones due to excessive deformations, and in blue the ones due to local defects.

Under all the considerations stated above, a full shell map was issued (Figure 8), representing the plates that needed replacement due to deformations/insufficient wall thickness and local defects.

It can be seen that around 82% of the tank shell is compromised and the entire shell should be replaced, for both technical/constructability and economic reasons. 

### 3.4. Tank Structural Health Monitoring (SHM)

**Figure 9:**
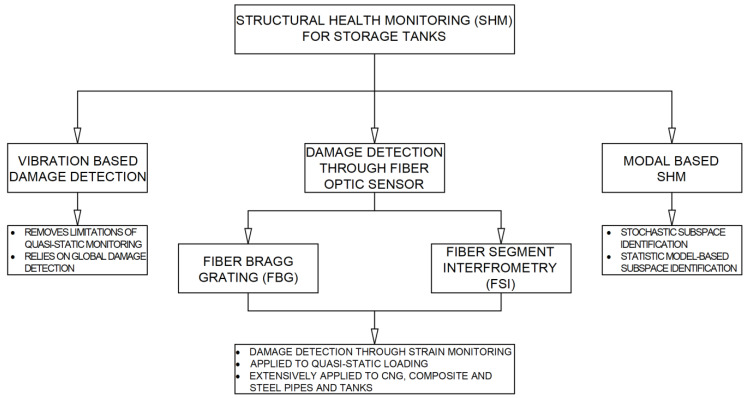
SHM techniques for storage tanks.

While non-destructive inspection detects any physical defects that have already occurred, structural health monitoring (SHM) can detect overstressed areas of tank components that may lead to future defects. To investigate structural integrity, a tangible structural health monitoring (SHM) technology is entailed for the health management of hydrogen storage tanks (Figure 9). For this task, flexible transducers are highly used within sensing systems to maximize the SHM method’s efficiency [27].

The most commonly used techniques used in the SHM of tanks are as follows:

- Strain-based monitoring is one of the most used passive SHM techniques for investigating storage tank flaws. The working principle is relatively simple. The structure strain directly reflects its deformations upon externally applied forces or any environmental changes (i.e., temperature) [7].
- Elastic wave-based SHM, which has an enhanced sensitivity for small-scale defects and can be successfully used for long-range inspection. Elastic wave-based structural health monitoring is based on the signal output of a transducer network induced by waves that propagate within the structure for determining the status of the investigated material. Based on the elastic wave type, this method can be divided into the following two categories: the active SHM category that uses ultrasonic-guided waves and another passive SHM that is based on acoustic emission.

A simpler approach for the SHM is proposed in [28].

The main methods for the SHM of storage tanks [27] are classified as follows:

- Strain gauge with resistive sensors for strain (damage detection, localization, and quantification are applied based on the sensing sheet), for stress (structural self-sensing for early damage prediction), and for pressure (self-sensing composites with both pressure and stress sensitivity).
- Strain gauge with capacitive sensors for pressure (pressure sensitivity with no responsiveness to strain and temperature) and for strain (a superior performance for high-elongation strain measurements, while sometimes complex electronic circuits are needed for capacitance acquisition and wireless application).
- Strain gauge with piezoelectric sensors for pressure (dynamic sensing for air pressure loading on shock tube pressure test) and for strain (a superior performance in crack opening detection and measuring the crack opening load).
- Optical inspection with Fiber Bragg grating sensors for strain (small size for embedded and temperature insensitivity on covered metal coatings) and for strain and temperature (a high accuracy for strain and temperature are verified).
- Optical inspection with distributed fiber optic sensors for temperature and humidity (fully distributed with a spatial resolution in the centimeter order and low measurement errors) and for strain and shape (continuous measurement using an embedded sensor in the host structure over the entire length).
- Electro-mechanical impedance with a piezoelectric transducer for damage (exciting the host structure and measuring its impedance response to detect void damage, as an actuator and sensor simultaneously and/or the parametric variation of the sensor would affect accuracy, stabilization, and signal repeatability).
- Ultrasonic-guided wave with a piezoelectric transducer or magnetostatic transducer to detect any flaw in the test medium.
- Acoustic emission with a piezoelectric sensor for any flaw source localization. Fatigue sub-stages using machine learning algorithms can be implemented.

### 3.5. Data Analysis and Predictive Modeling

**Figure 10:**
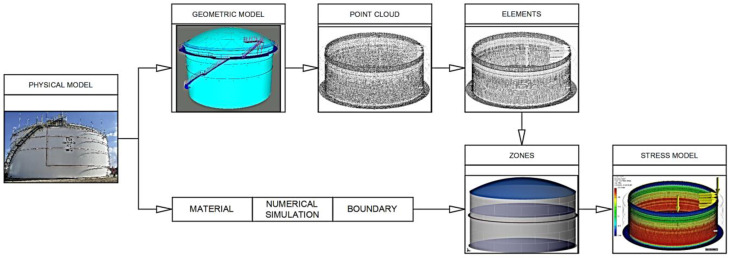
Typical Digital Twin model building process.

These methods were developed within recent decades and gained more and more popularity as they were systematically validated. As computer processing capabilities and software development increased exponentially, equipment condition assessment could be performed using a computer model of the existing equipment. A Digital Twin (DT) [29], as shown in Figure 10, is a virtual, digital model that replicates real-world entities or systems, enabling them to undergo simulation, monitoring, and analysis in a digital environment [29].

The tank model is formulated following the tank inspection/measurement and using its real dimensions. Also, 3D measurements (point clouds) are used, but the final model should be validated by an experienced engineer. It is very important that the 3D model takes into account the deformations identified by 3D scanning and the thicknesses of the plates that are input manually following the ultrasonic wall thickness measurements, or automatically, in case the bottom/shell/roof plates, which were continuously scanned (i.e., by SLOFEC or similar methods). Any other appurtenances (like reinforcing rings, central pillars, etc.) might need to be manually modeled.

It is recommended that the roof supporting structure, if any, is modeled and assessed separately, since its behavior is different from the other tank elements and it could be difficult to connect the beam elements to the shell elements, so the final results might not reflect the real situation of the elements. Moreover, for a steel structure, usually another design/verification code should be used (i.e., in the European Union, Eurocode governs the steel structure design).

After modeling completion, the model should be filled with loads for each considered case (operating, wind, seismic, etc.) and their combinations according to applicable standards.

For each case, simulations are performed, and the results should be included in a report.

In some particular cases, the output data from SHM systems can be automatically processed and loaded as inputs in the model by using AI technology [30].

### 3.6. Comparative Analysis of Condition Assessment Techniques

The selection of a technique for condition assessment (Table 5) is based on the equipment’s state, importance, and even economic criteria. For example, if an old tank is subjected to a visual inspection and is in a very bad shape, with advanced corrosion, a loss of content, and damaged components, there is no need for a complex condition assessment. It is clear that the damaged parts, if not the entire tank, need to be replaced, and an experienced engineer will decide this in their report. There could be situations when a thorough inspection of a small tank using the most advanced techniques described in this article would be more costly than a new tank itself. On the other hand, if a large tank is inspected and, for example, local corrosion and plate thinning are found, together with localized corrosion due to the internal coating failure of some roof structure members, but the other tank components are in a good condition, then a full inspection followed by a condition assessment and some minor reports could bring the tank back into safe operation condition for another 15 or 20 years with for a fraction of the cost of a new tank.

## 4. Standards and Regulations

### 4.1. Relevant International Standards

The main governing standards for storage tank condition assessments are [3,4,31,32], each treating, from a similar perspective, the fitness for service, inspection, and repairment of equipment (in this case, storage tanks).

It is mandatory for storage tanks to be inspected and assessed in accordance with the specifications of standard [3] set by the American Petroleum Institute (API). There have been an increasing number of reported cases of leakages, failures, fires, and explosions involving storage tanks in recent years, so safety and design concerns have become increasingly important. Several parameters are taken into consideration when inspecting/assessing the technical state of storage tanks, mainly the current state of the tank components (bottom, shell, roof, etc.) along with process parameters and safety, mechanical, civil, structural, and instrumental factors. Tank size, flow capacity, temperatures/pressures, and stored fluid characteristics are some of the most important process considerations. A number of calculations are detailed in [3] to ensure that tanks are able to withstand mechanical loads such as wind, earthquakes, internal fluid pressures, and hydrostatic/hydrodynamic loads [33].

### 4.2. National Regulations and Industry-Specific Guidelines

There are numerous countries, especially in the European Union and Asia, that have dedicated standards for storage tank design/inspection, but none of them are as detailed and proven as [3,4,31,32].

The use of local standards is required sometimes by local authorities or clients via tender books or requisitions, even if their applicability is limited. In this case, it is the engineer’s responsibility to carefully perform the inspection and condition assessment, taking into consideration the client and local regulatory indications, but always respecting the most stringent requirements of the dedicated standards mentioned above. In such situations, it is worth mentioning the European standards [6,24].

### 4.3. Challenges in Complying with Diverse Standards and Regulatory Bodies

The most challenging task in preparing a condition assessment that complies with the client and local authorities of non-USA countries is harmonizing both assessment/calculation methods and results and justifying selecting the most stringent situation.

There are two main differences here. One is in the input, which is somehow different, for example, in the USA, where seismic behavior is well-established in areas, while in other countries, it is characterized by certain factors like seismic acceleration, corner periods, etc. The other is calculation methods, which are fundamentally different in most standards.

In order to further exemplify this, there are two European norms that are fundamentally different and are used for the same purpose, namely, the design of aboveground storage tanks, as follows:

- The limit states method, adopted by [34]. This method addresses the Service Limit State (SLS), which concerns structural failure, and the Ultimate Limit State (ULS), which concerns structural integrity at the minimum operational level.
- The allowable stress method, adopted by [6,26]. The standard [6] proposes the analysis of the following two states in the case of an earthquake: operational (Operating Basis Earthquake, OBE) and safe shutdown (Safe Shutdown Earthquake, SSE), comparable to those in Eurocodes. The API standard defines three states, with the first being the operational state during an earthquake (Operating Level Earthquake, OLE), the second being the contingency state (Contingency Level Earthquake, CLE), and the third involving the occurrence of seismic aftershocks (Aftershock Level Earthquake, ALE).

In these situations, the best practice is to impose the dedicated standards for tank condition assessment, which are mentioned in Section 4.2, and additionally to use local standards as a reference.

## 5. Challenges and Limitations

Even if the inspection and condition assessment techniques for storage tanks have evolved over the years, there are some key areas that can create difficulties for some activities. Here, the following can be mentioned: ultrasonic thickness measurements that cannot be accessed in some areas by robots or drones, such as the underside of the roof plates between some structural steel profiles, welding inspection activities that cannot be performed from a distance, and personal access needs to be provided at certain locations. This access usually involves the use of scaffolding, which has significant impacts on both inspection cost and schedule.

There are also situations when ultrasonic scanning robots can be used, but there are limitations due to lack of access. It is also worth mentioning the situation when the object of the condition assessment is a small tank, for which contracting certain specialized companies to automatically scan and measure wall thicknesses would simply not be feasible from an economical point of view. A skilled API inspector using classical measuring methodology could successfully perform this task at a fraction of the cost.

In some of these situations, old-fashioned methods are imposed, as they are proven and remain the only means that can be used.

## 6. Emerging Technologies and Trends

It is well known that, in the last few years, a lot of software packages have been developed in order to help and assist engineers in the design, fabrication, construction, and condition assessment of equipment.

There is no complete software solution to fully perform a tank condition assessment, so different parts of the tank are verified using different software and standards (in this case, the loads and connections should be transferred from one part to another). For example, for a roof steel structure evaluation, the roof plates and any other roof structures are considered as dead weight, while the beams-to-shell connections are considered as supports. This is one of the several aspects that the engineer performing the assessment needs to consider.

But things have gone even further, especially regarding the automation of design and inspection/condition assessment for storage tanks. To be more precise, if engineers had to hand model equipment while considering wall thickness measurements before, the newest technologies have improved this process by combining different inspection techniques and automatically creating a Digital Twin of the tank [35]. The combined technologies include the following:

- Laser scanning, also known as Light Detection and Ranging (LIDAR)—for dimensional and deformation scanning;
- SLOFEC scanning for shell and bottom plate wall thickness measurements, which can be used up to 30 mm [36];
- PMI (Positive Material Identification) and mechanical strength tests to identify the materials for different tank components;
- Optionally strain gauges can be used on different elements, but since the tank is not pressurized equipment or subjected to a cyclical load, these have a limited applicability, only for highly deformed or thinned areas;
- Three-dimensional modeling and parametrization software packages, like Ansys, Autodesk Inventor, Fusion, etc.

Using the above techniques and a dedicated software package, an experimental engineer can significantly improve the time and accuracy of condition assessment.

Also, even if there is no available dedicated software, there are some platforms that allow engineers to input the measured data, and these platforms can automatically perform assessments according to the dedicated standards and output a full report regarding the tank state. An example of such a software platform is Black Wolf Inspection Technologies.

For specific unstandardized applications, parametrical models of equipment can be prepared for later usage according to the data taken from field measurements. This can be applied, for example, for double shell tanks [37], which are not specifically treated by any standard or recommended practice. Most 3D modeling software packages support parametric modeling, and an experienced engineer can prepare a typical model that can be exported in analysis software. One dedicated storage tank modeling software (parametric, based on Autodesk Inventor Engine with a proprietary software interface) is SEG by SECAD Solutions.

## 7. Future Directions and Recommendations

The latest technological advances in robotics, machine learning, and artificial intelligence (AI) have allowed for the further automation of both inspection techniques and the interpretation of results.

Along with these two inspection techniques, equipment monitoring can also be achieved remotely with Internet of Things (IoT) [38,39] applications, and combining these with AI-powered algorithms to enable predictive maintenance can ultimately increase equipment lifetime while decreasing downtime for maintenance. IoT sensors can measure and transmit real-time data regarding corrosion rate and strain, which are very important for tank monitoring.

Regarding the measurement of tank components, automated robotic inspection devices—wall scans (crawlers)—can be used [40,41,42,43,44] for wall thickness measurements, while automated drones can be used for inaccessible areas inspections, spot wall thickness measurements, and NDT.

One of the most important consequences, besides operational safety, is the overall maintenance cost and equipment availability, which can be significantly improved by implementing the latest technologies.

The downside of the implementation of these technologies is the lack of standardization or legislation. Unfortunately, often, the standardization process does not manage to keep pace with the latest technological evolution, so companies can be skeptical about implementing some new techniques.

## 8. Conclusions

Tank condition assessment activities are governed by the well-known standards and recommended practices mentioned in this article, with the steps for performing such assessments being as follows:

- Performing the inspections, measurements, and NDT activities;
- Performing the necessary calculations;
- Issuing the final condition assessment report along with the eventual revamping work needed to be performed to bring the equipment into a safe operating state.

Even if the steps remain the same, efforts have been made to both improve the accuracy and increase the efficiency of inspection activities and to further increase the automation of calculations and data analysis to obtain a model as close as possible to the reality in the field. The accuracy of an assessment report is proportional to the quality of data from the inspection. Also, the efficiency of the method (in terms of time) is proportional to the degree of automation of the evaluation process, and this can be increased by making use of the latest technological developments, like AI, IoT, etc.

Breakthroughs in computer development have impacted all markets, and equipment assessment activities can benefit from the latest emerging technologies. This impact can be qualitative, by increasing the assessment accuracy, and quantitative, by positively improving the activities’ schedule. This is the same for cost, which can be improved in several manners, among which the following can be mentioned:

- A shorter time for inspection activities by using scanners/robots;
- Eliminating the necessity of scaffolding by using robotic systems/drones;
- Decreasing human activities by using automated systems;
- Limiting the manhours needed for data analysis by using advanced AI algorithms, dedicated software packages, and machine learning;
- Predictive maintenance that can be automatically performed by the installation of sensors;
- Increasing equipment availability;
- Significantly decreasing the risk of accidents or environmental pollution [45,46,47,48,49];
- In some cases, all these measures can make a standing point in insurance evaluation.

The main goal of this paper is to propose a condition assessment algorithm that can be accepted in the EU and meet the EU design norms, but, in the meantime, take advantage of the dedicated API standards that are based on extensive studies. The key of a comprehensive condition assessment is the engineer’s capability to select the best applicable inspection techniques and analysis methods (within the standards requirements) for the equipment technical state, while also having in mind cost and schedule considerations.

## Figures and Tables

**Table 1:** Tank M6 wall thickness assessment according to [3].

Course	Average Measured Thickness *t*_1_ (mm) *	Absolute Minimum Measured Thickness *t*_2_ (mm)	Minimum Allowable Thickness *t_min_* + Corrosion Allowance (mm)	Course Status **
1	18.00	18.00	13.85	Course OK
2	14.32	13.10	11.80	Course OK
3	12.47	11.50	9.18	Course OK
4	9.75	7.80	7.44	Course OK
5	9.14	8.30	6.54	Course OK
6	6.65	6.30	6.54	Course OK
7	7.85	7.40	6.54	Course OK
8	5.92	5.60	6.54	Course Fail
9	5.54	5.00	6.54	Course Fail

* Value *t*_1_ is considered the average of the entire shell course. ** According to [3]: The criteria for continued operation is as follows: (i) the value *t*_1_ shall be greater than or equal to *t_min_*, subject to verification of all other loadings listed; (ii) the value *t*_2_ shall be greater than or equal to 60% of *t_min_*; and (iii) any corrosion allowance required for service until the time of the next inspection shall be added to *t_min_* and 60% of *t_min_*.

**Table 2:** Tank M6 wall thickness values assessment according to [6].

Course	Minimum Measured Thickness (mm)	Minimum Resistance Thickness (mm)	Course Status	New Plates Thickness for Repairment *
1	18.00	14.11	Course OK	16.00
2	13.10	12.70	Course OK	14.00
3	11.50	11.38	Course OK	13.00
4	7.80	10.06	Course Fail	12.00
5	8.30	8.73	Course Fail	10.00
6	6.30	7.38	Course Fail	9.00
7	7.40	6.03	Course OK	8.00
8	5.60	4.67	Course OK	7.00
9	5.00	4.54	Course OK	7.00

* These are the wall thicknesses for the new plates if the minimum measured thickness of the existing ones does not correspond.

**Table 3:** Tank M6 wall thickness values assessment according to [24].

Course	Vertical Distance from the Bottom of the *j* Course to the Liquid Level (m)	Reduced Vertical Distance from the Bottom of the *j* Course to the Liquid Level (m)	Calculated Stress (MPa)	Maximum Allowable Stress (MPa)	Course Status
1	12.97	12.97	119.508	133.200	Course OK
2	11.47	11.17	148.361	133.200	Course Fail
3	10.07	9.77	151.625	133.200	Course Fail
4	8.67	8.37	212.771	133.200	Course Fail
5	7.25	6.95	162.660	133.200	Course Fail
6	5.82	5.52	189.295	133.200	Course Fail
7	4.39	4.09	111.700	133.200	Course OK
8	2.94	2.64	108.178	133.200	Course OK
9	1.49	1.19	58.568	133.200	Course OK

**Table 4:** Hoop stress assessment for Tank M6 shell.

Course	Hoop Stress (kPa)	Allowable Soop Stress (kPa)	Course Status
1	96,897.59	111,037.49	Course OK
2	119,518.49	111,037.49	Course Fail
3	117,506.52	111,037.49	Course Fail
4	156,177.45	111,037.49	Course Fail
5	110,271.21	111,037.49	Course OK
6	113,539.73	111,037.49	Course Fail
7	54,085.77	111,037.49	Course OK
8	N/A *	111,037.49	Course OK
9	N/A *	111,037.49	Course OK

* For the last two courses, hoop stress value was negligible and was not computed.

**Table 5:** The characteristics of different techniques used for condition assessment.

Method	Advantage	Disadvantage	Observation
Visual inspection	Reduced equipment cost	Highly qualified personnel required	Mandatory for condition assessment
NDT	N/A	High equipment cost, highly qualified personnel required	Mandatory for condition assessment
Analytical	Low cost for implementation	Highly qualified engineers required	Mandatory for condition assessment
SHM	Quality input for assessment	High equipment cost, highly qualified engineers are required, relatively expensive	
Data Analysis	Precise output, detailed analysis	Highly qualified engineers required	
Predictive modeling	Precise output, detailed analysis, defect prediction	Highly qualified engineers are required, specific processing equipment required	

## Data Availability

The original contributions presented in this study are included in the article. Further inquiries can be directed to the corresponding authors.

## Abbreviations

The following abbreviations are used in this manuscript:

NDT	Non-destructive testing
FEA	Finite Element Analysis
AST	Aboveground storage tank
API	American Petroleum Institute
EEMUA	Engineering Equipment and Materials Users Association
LIDAR	Light Detection and Ranging
SLOFEC	Saturated Low-Frequency Eddy Current
UT	Ultrasonic testing
MPT	Magnetic particle testing
AE	Acoustic emission (testing)
RT	Radiographic testing
PT	Dye penetrant test
LCO	Light Cycle Oil
IFR	Internal floating roof
SHM	Structural health monitoring
DT	Digital Twin
SLS	Service Limit State
ULS	Ultimate Limit State
OBE	Operating Basis Earthquake
SSE	Safe Shutdown Earthquake
OLE	Operating Level Earthquake
CLE	Contingency Level Earthquake
ALE	Aftershock Level Earthquake
PMI	Positive Material Identification
AI	Artificial intelligence
IoT	Internet of Things
HAZ	Heat-affected zone
EU	European Union
CA	Corrosion allowance
MDMT	Minimum Design Metal Temperature
